# Higher diagnostic value of metagenomic next-generation sequencing in acute infection than chronic infection: a multicenter retrospective study

**DOI:** 10.3389/fmicb.2024.1295184

**Published:** 2024-01-29

**Authors:** Anjie Yao, Jiale Wang, Qintao Xu, Binay Kumar Shah, Kai Sun, Feng Hu, Changhui Wang, Shuanshuan Xie

**Affiliations:** ^1^Department of Respiratory Medicine, Shanghai Tenth People's Hospital, Tongji University School of Medicine, Shanghai, China; ^2^School of Medicine, Tongji University, Shanghai, China; ^3^College of Medicine, Jinggangshan University, Ji'an, China; ^4^Department of Respiratory Medicine, ChongMing Branch of Shanghai Tenth People's Hospital, Tongji University School of Medicine, Shanghai, China; ^5^Department of Respiratory and Critical Care Medicine, Tongren Hospital, Shanghai Jiao Tong University School of Medicine, Shanghai, China

**Keywords:** metagenomic next-generation sequencing, bronchoalveolar lavage fluid, acute infection, chronic infection, diagnostic

## Abstract

**Background:**

The aim of this study is to compare the diagnostic value of metagenomic next-generation sequencing (mNGS) vs. conventional culture methods (CM) in chronic infection and acute infection.

**Methods:**

We retrospectively analyzed the bronchoalveolar lavage fluid (BALF) of 88 patients with acute infection and 105 patients with chronic infection admitted to three hospitals from 2017 to 2022.

**Results:**

The results showed that the sensitivity and specificity of mNGS were higher than those of CM. The number of patients who changed the antibiotic treatment in the mNGS positive group was larger than that of patients in the mNGS negative group in both the acute infection group (60.5 vs. 28.0%, *P* = 0.0022) and chronic infection group (46.2 vs. 22.6%, *P* = 0.01112). High levels of temperature (OR: 2.02, 95% CI: 1.18–3.70, *P*: 0.015), C-reactive protein (CRP) (OR: 15, 95% CI: 2.74–280.69, *P*: 0.011), neutrophil count (OR: 3.09, 95% CI: 1.19–8.43, *P*: 0.023), and low levels of lymphocyte count (OR: 3.43, 95% CI:1.26–10.21, *P*: 0.020) may lead to positive mNGS results in the acute infection group while no significant factor was identified to predict positive results in the chronic infection group.

**Conclusion:**

mNGS could provide useful guidance on antibiotic strategies in infectious diseases and may be more valuable for the diagnosis and treatment of acute infection vs. chronic infection.

## Introduction

Respiratory tract infection represents the most prevalent infectious disease and constitutes a formidable clinical challenge due to its diverse etiologies. Clinically, ~19–62% of respiratory tract infection cases are etiologically unclear. Whether respiratory-borne infections were due to bacteria, viruses, or fungi, they have witnessed a distressing surge in both their incidence and the subsequent mortality on a global scale (Jin et al., 2022). Our ability to treat infections is jeopardized by antimicrobial use and resistance. The devastating result of infection emphasizes how crucial early diagnosis and effective antibiotic treatment are for diseases. Microbial culture, antigen/antibody assays, and polymerase chain reaction (PCR)-based nucleic acid detection are the primary components of conventional molecular testing for pathogen identification. Complete identification of fastidious organisms is often reserved for isolates in pure culture. However, it is a time-consuming and arduous technique that will take several days to complete (Li et al., 2020). In addition, the sensitivity of the conventional culture methods is heavily influenced by the duration of the infection and whether or not the patient has already received antibiotic treatment. Antigen/antibody assays have a narrow range of applications, and the results are often impacted by the threshold. Despite its excellent specificity and sensitivity, PCR still requires pathogen prediction in order to create the appropriate primers (Xiao et al., 2023).

The limitations of CM frequently result in delayed or incorrect diagnosis and even improper antibiotic administration. As a result, timely and precise identification of unknown pathogenic microorganisms is crucial for guiding clinical decision-making regarding diagnosis and therapy. Metagenomic next-generation sequencing (mNGS) is a new pathogen detection method with excellent effectiveness and has grown steadily in healthcare settings due to excellent effectiveness, a broad pathogen spectrum, and enhanced sensitivity. Theoretically, mNGS performs unbiased, meticulous high- throughput sequencing of the total DNA or RNA content of nearly all recognized pathogens, including bacteria, fungi, viruses, *Mycobacterium tuberculosis*, parasites, and atypical pathogens, and the sequence data obtained are then compared with databases (Gu et al., 2021). In addition, previous antibiotics have an effect on the diagnostic accuracy of mNGS compared with CM (Lv et al., 2023).

An updated report of Global Burden of Disease 2019 shows that chronic respiratory diseases are the third leading cause of death with mortality of 4.0 million and prevalence of 454.6 million cases globally (GBD 2019 Chronic Respiratory Diseases Collaborators, 2023). In recent years, the utility of mNGS for the detection and diagnosis of respiratory tract infections has been studied. However, the different impacts of mNGS of non-sterile body fluids such as bronchoalveolar lavage fluid (BALF) on the diagnosis and prognosis of patients with acute infection and chronic infection remain controversial. In this multicenter retrospective study, we explored the diagnostic value of mNGS in the early detection of microorganisms by comparing acute and chronic infections in BALF samples, hoping that the results could help diagnosis and treatment of patients with acute and chronic infections.

## Methods

### Study design and data collection

**Figure 1:**
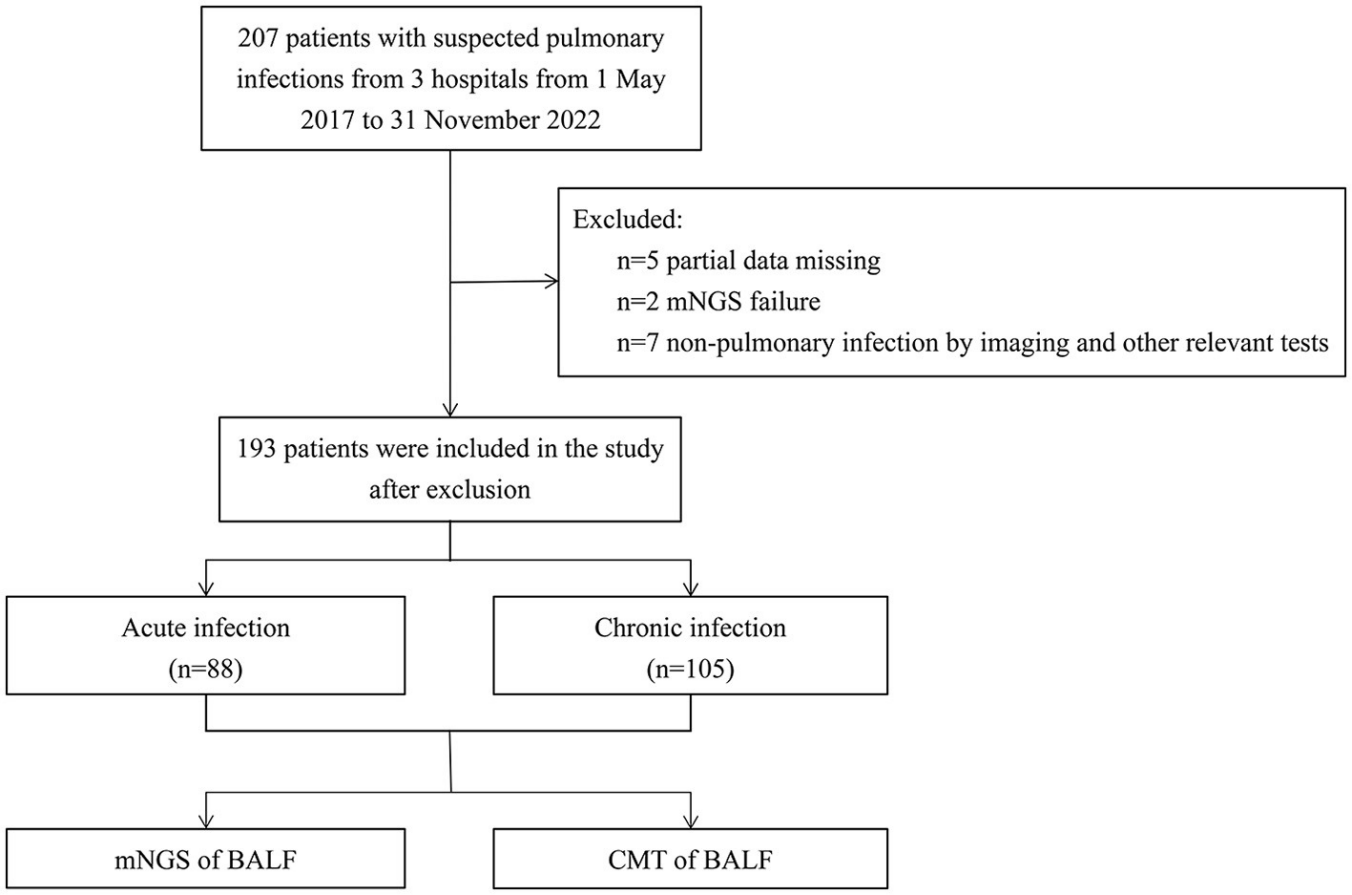
Flow diagram. mNGS, metagenomic next-generation sequencing; CMT, culture methods.

This multicenter retrospective study analyzed 193 patients with suspected pulmonary infection who were admitted from May 2017 to November 2022 at three hospitals: the General institute of Shanghai Tenth People's Hospital, the Chongming Branch of Shanghai Tenth People's Hospital, and the Tongren Hospital. The study was approved by the Ethics Committee of the Tenth People's Hospital of Tongji University. The recruitment process is shown in Figure 1. Exclusion criteria were: (1) patients younger than 18 years, pregnant women, and psychiatric patients; (2) patients with non- pulmonary infection as shown by imaging and other relevant tests; (3) patients with partial data missing or mNGS failure. Based on the course of disease, radiological images, and laboratory test results, the patients were classified into an acute infection group (*n* = 88) and a chronic infection group (*n* = 105). Patients in the acute infection group had pulmonary infection for less than a month and new infections on imaging of chest computed tomography (CT), and patients in the chronic group had pulmonary infection for more than a month with no presence of new infections on chest CT imaging. CM and mNGS results, patient clinical characteristics, blood test results, antibiotic treatments, length of hospital stay, survival outcomes during hospitalization, and the clinical outcome of each patient were collected and analyzed.

### Culture method

BALF was collected from patients with suspected pneumonia by bronchoscopy within 72 h after admission to the hospital, and the collected BALF specimen was sent to the Department of Clinical Laboratory of Shanghai Tenth People's Hospital for mNGS and CM examination. BALF was qualified according to the following conditions: no airway secretion in the BALF, 40% recovery with more than 95% cell survival, 10% erythrocytes (excluding trauma/hemorrhagic factors), and 3–5% epithelial cells and undistorted intact smear cells (Wang et al., 2019). Blood agar, chocolate agar, and MacConkey agar plates were used for bacterial culture at 35°C and 5% CO2 concentration. Roche medium was used for mycobacterial culture, and Sabouraud Dextrose Agar was used for fungi at 37 and 25°C, respectively.

### Metagenomic next-generation sequencing

BALF was collected by bronchoscopy, and mNGS was performed after admission or within 72 h of disease onset. After obtaining BALF samples, mNGS was performed. A standard operating procedure of the DNA-based mNGS method was developed for the diagnosis of pathogens. In brief, 1 ml of sample was centrifuged at 12,000 × g for 5 min to collect the pathogens and human cells. Next, 50 μl of precipitate underwent depletion of host nucleic acid using 1 U of Benzonase (Sigma) and 0.5% Tween 20 (Sigma) and incubated at 37°C for 5 min. Terminal buffer (400 μl) was added to stop the reaction. Then, the quantified unique DNA fragments (named UMSI) were spiked for each sample as an identity and internal control, which were PCR products of *Oryza sativa* 400–600 bp in length. In total, 600 μl of the mixture was transferred to new tubes containing 500 μl of ceramic beads for bead beating using a Minilys personal TGrinder H24 homogenizer (catalog number OSE- TH-01; Tiangen, China). Then, nucleic acid from 400 μl of pretreated samples was extracted and eluted in 60 μl of elution buffer using a QIAamp UCP pathogen minikit (catalog number 50214; Qiagen, Germany). The extracted DNA was quantified using a Qubit double-stranded DNA (dsDNA) high-sensitivity (HS) assay kit (catalog number Q32854; Invitrogen, USA).

In total, 30 μl of the eluate was used to generate libraries using the Nextera DNA Flex Kit (Illumina, San Diego, CA, USA), according to the manufacturer's instructions. Fragmentation and tagmentation of the DNA were performed using the bead-linked transposome. After completion of post-tagmentation cleanup, the tagmented DNA was amplified; the thermocycling parameters were as follows: 68°C for 3 min and 98°C for 3 min, followed by 18 cycles of 45 s at 98°C, 30 s at 62°C, and 2 min at 68°C, before a final minute at 68°C. Dual indexing was conducted by employing the IDT for Illumina DNA/RNA UD indexes (catalog number 20027213). Purification and size selection were carried out following the double-sided bead purification procedure. A Qubit dsDNA HS assay kit was used to measure the library concentration. Library quality was assessed with an Agilent 2100 Bioanalyzer (Agilent Technologies, Santa Clara, CA, USA) using a high-sensitivity DNA kit. The library was prepared by pooling 1.5 pM concentration of each purified sample equally for sequencing on an Illumina NextSeq 550 sequencer using a 75-cycle single-end sequencing strategy.

For bioinformatics analysis, Trimmomatic was used to remove low-quality reads, adapter contamination, duplicate reads, and reads shorter than 70 bp. Low-complexity reads were removed by Kcomplexity using default parameters. The human sequence data were identified and excluded by mapping a human reference genome (hg38) using SNAP v1.0beta.18. To construct the microbial genome database, pathogens and their genomes or assemblies were selected following the Kraken2 criteria for selecting representative assemblies for microorganisms (bacteria, viruses, fungi, protozoa, and other multicellular eukaryotic pathogens) from the NCBI Assembly and Genome databases (https://benlangmead.github.io/aws-indexes/k2). Microbial reads were aligned to the database using Burrows-Wheeler Aligner software. We defined that reads with 90% identity of reference were mapped reads. In addition, reads with multiple locus alignments within the same genus were excluded from the secondary analysis. Only reads mapped to the genome within the same species were considered.

We normalized the sequencing reads RPTM to eliminate the errors caused by various sequencing depths among samples. To establish the optimal threshold value for the >10 microbes with culture isolates, samples spiked with microbes were defined as positive samples, while negative control (NC) was defined as the negative sample. Receiver operating characteristic curves were plotted for each target species using these samples. The parameter resulting in the highest area of AUC was considered the positive cutoff value for this species. For microorganisms without culture isolates, the RPTM mean value and standard deviation of this microorganism were calculated, and the RPTM [mean + 2 standard deviations (SD)] was set as a positive cutoff value.

The clinical reportable range (CRR) for pathogens was established according to the following three references indicated in a previous study: (i) the Johns Hopkins ABX Guide (https://www.hopkinsguides.com/hopkins/index/Johns_Hopkins_ABX_Guide/Pathogens), (ii) Manual of Clinical Microbiology, and (iii) clinical case reports or research articles published in peer-reviewed journals. All microbes that exceeded the threshold of mNGS were classified into three categories: (i) probable (BALF mNGS-based results were within the CRR and concordant with the clinical and radiologic results; the RPTM was significantly higher than the positive cutoff value, and the abundance was obviously higher than that of other species of the same genus), (ii) possible (the microbe has pathogenic potential, but an alternate explanation is more likely), and (iii) unlikely (the microbe cannot cause pneumonia).

To monitor the sources of potential contamination, both NC and sterile deionized water, which served as non-template controls, were prepared in parallel with other samples in each batch. In addition, we used sterile cotton swabs dipped in sterile deionized water to wipe the surfaces of the centrifuge and biosafety cabinet, to generate the background microorganism list in our laboratory.

### Golden standard based on clinical compound diagnosis

Two respiratory physicians with expertise in respiratory management independently reviewed the medical records and the results of CM and mNGS of all patients. First, they determined whether the patient had a lung infection; second, they identified causative pathogens based on the patient's complaints, clinical presentation, laboratory findings, imaging presentation, and microbiological investigations (including CM and mNGS). Finally, they made a decision on the treatment regimen or adjusted the treatment regimen used. Disagreements between the two physicians regarding the causative pathogen were resolved through in-depth discussion until the consensus was reached, or if consensus could not be reached, another respiratory physician with a higher professional title would be consulted.

### Criteria for mNGS positive results

The data were filtered to delete the reads with low quality, containing sequencing adapters to obtain the clean reads. After subtracting human host sequences in the clean reads, the remaining non-human sequences were compared with the microbial genome database. If the presence of a pathogenic organism met with any of the following criteria, the mNGS result was judged to be positive [RPM (reads per million) = Mapped reads number × 106/total sequencing reads; SDSMRN (stringently mapped reads number) = mapped reads number × 20 × 106/total sequencing reads]: (1) Bacteria: RPMsample/NTC ≥ 10, SDSMRN ≥3; (2) Fungi: RPMsample/NTC ≥ 1, SDSMRN ≥ 3; (3) RNA virus: RPMsample/NTC ≥ 1, SDSMRN ≥ 1; (4) DNA virus: RPMsample/NTC ≥ 1, SDSMRN ≥ 3 (Liang et al., 2023).

### Statistical analysis

SPSS 25.0 and R language (4.3.1) statistical software were used for data processing and analysis and graphing. Normally distributed data were expressed as *x* ± *s*; Pearson's chi-square test was used to compare the differences of categorical variables between different groups; logistic regression analysis was used to explore the risk factors associated with acute and chronic lung infections; and COX proportional risk regression was used to explore the effects of different factors on survival outcomes. All tests were two-tailed, and *P* < 0.05 was considered statistically significant.

## Results

### Characteristics of patients

As presented in Table 1, no significant difference was observed in the baseline characteristics between acute and chronic infection groups. The proportion of older male patients aged 60–75 years was 42.0% in the acute infection group and 46.6% in the chronic infection group. The proportion of patients complicated with other diseases (such as hypertension, diabetes, cardiac disease, stroke, and tumors) was 81.8% in the acute infection group and 65.7% in the chronic infection group, with hypertensive diseases predominating, 21.6% in the acute infection group, and 22.9% in the chronic infection group. The detection rates of CM were 22.7% in the acute infection group and 35.2% in the chronic infection group. The detection rates of mNGS were 43.2% in the acute infection group and 49.5% in the chronic infection group, which were all higher than those of CM. However, the overall detection rates were not significantly influenced by the disease characteristics in either CM or mNGS.

**Table 1:** Baseline characteristics of patients between acute infection group and chronic infection group.

**Characteristics**	**Acute infection (*n* = 88)**	**Chronic infection (*n* = 105)**	***P*-value**
Sex			0.488
Female	37 (42.0%)	39 (37.1%)	
Male	51 (58.0%)	66 (62.9%)	
Age			0.362
< 45	18 (20.4%)	12 (11.4%)	
45–60	22 (25.0%)	27 (25.7%)	
60–75	37 (42.0%)	49 (46.6%)	
≥75	11 (12.5%)	17 (16.1%)	
Comorbidities			0.284
No	16 (14.7%)	36 (28.1%)	
Hypertension	19 (17.4%)	24 (10.9%)	
Diabetes	12 (11.0%)	9 (7.0%)	
Coronary heart disease	8 (7.3%)	8 (6.3%)	
Arrhythmia	3 (2.8%)	2 (1.6%)	
Stroke	7 (6.4%)	6 (4.7%)	
Tumor	6 (5.5%)	7 (5.5%)	
Autoimmune disease	1 (0.9%)	4 (3.1%)	
Others	37 (33.9%)	32 (25.0%)	
CM			0.058
Negative	68 (77.3%)	68 (64.8%)	
Positive	20 (22.7%)	37 (35.2%)	
mNGS			0.379
Negative	50 (56.8%)	53 (50.5%)	
Positive	38 (43.2%)	52 (49.5%)	

mNGS, metagenomic next-generation sequencing; CM, culture methods.

### mNGS is superior to CM for diagnosis of infections

As shown in Table 2, mNGS was more sensitive than CM (42.3 vs. 25.5%), and its specificity was higher than that of CM (10.7 vs. 7.1%). The positive predictive values (PPV) of the two groups were 96.3 and 95.5%, and the negative predictive values (NPV) were 22.3 and 17.5%, respectively.

**Table 2:** Comparison between mNGS and culture methods in all the patients.

**Test**	**Sensitivity**	**Specificity**	**PPV**	**NPV**
mNGS	47.27%	10.71%	96.3%	22.32%
CM	25.45%	7.14%	95.45%	17.45%

mNGS, metagenomic next-generation sequencing; CM, culture methods; PPV, Positive predictive value, the percentage of patients with disease determined by gold standard to the number of mNGS positive patients; NPV, negative predictive value, the percentage of patients without disease determined by gold standard to the number of mNGS negative patients.

**Figure 2:**
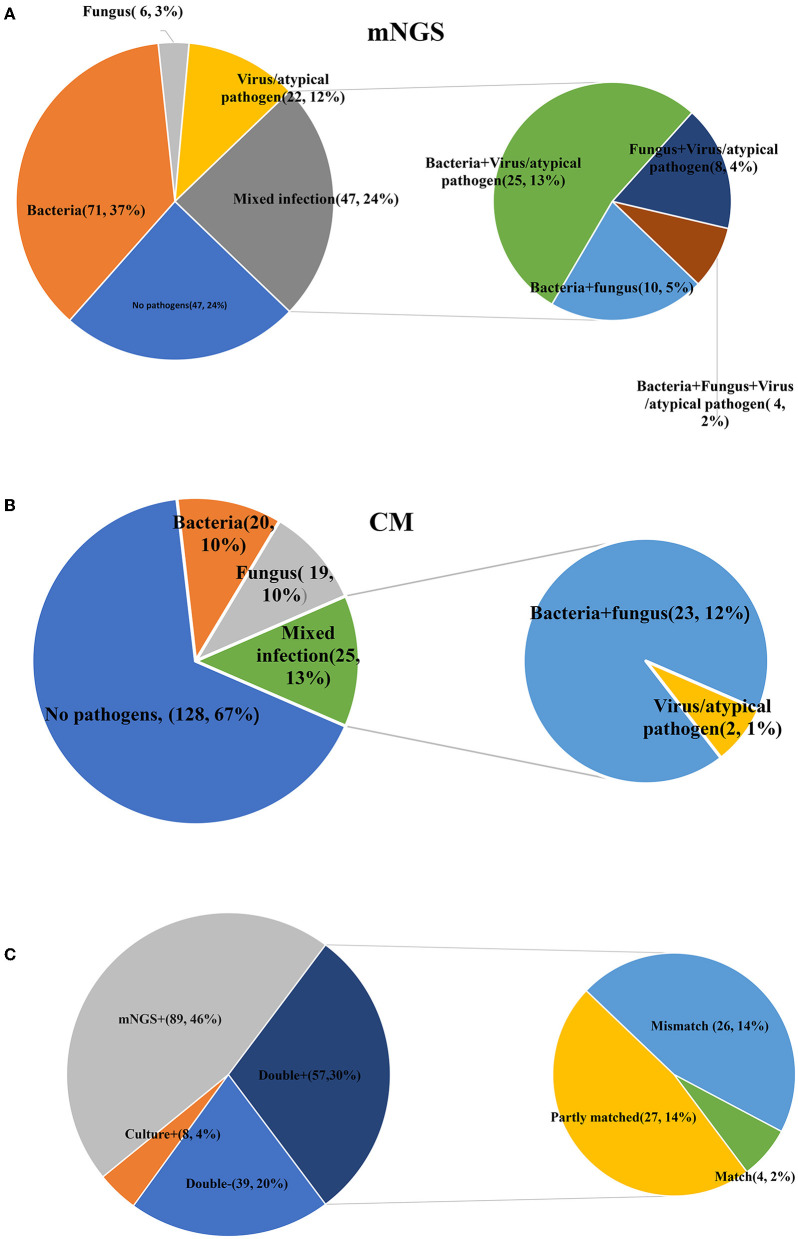
Comparisons between CM and mNGS in all patients. **(A)** The detection results of mNGS; **(B)** The detection results of CM; **(C)** The matched results between CM and mNGS. mNGS, metagenomic next-generation sequencing; CM, culture methods.

**Figure 3:**
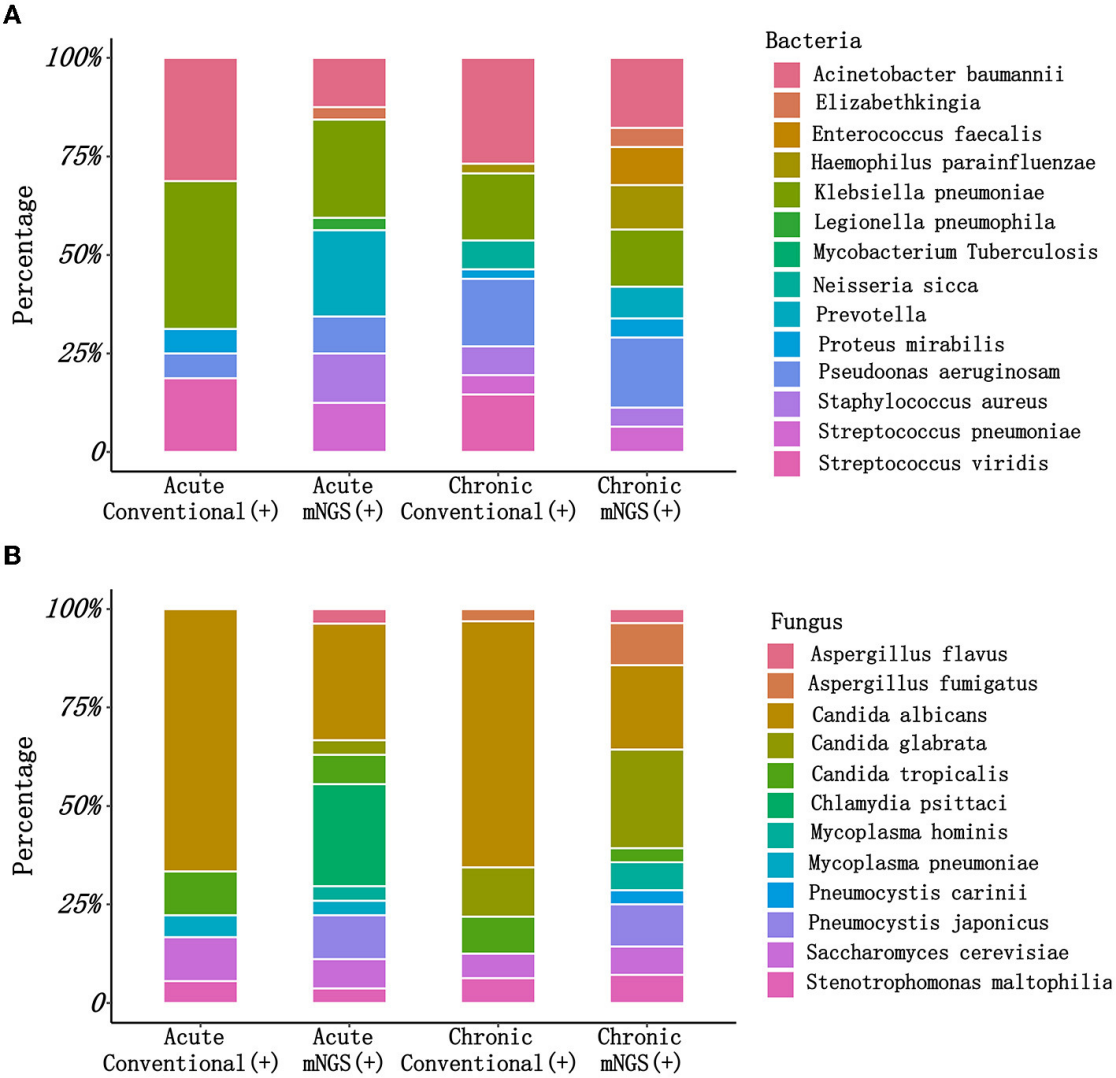
The pathogen types detected by CM and mNGS in different groups. **(A)** The bacteria types detected by CM and mNGS between acute infection group and chronic infection group; **(B)** The fungus types detected by CM and mNGS between acute infection group and chronic infection group. mNGS, metagenomic next-generation sequencing; CM, culture methods.

Additionally, there were substantial disparities in the results obtained from CM and mNGS. mNGS had the potential to significantly alter the distribution of detected pathogens, including many pathogens that could not be identified by CM. As shown in Figure 2, mNGS was dominated by bacterial infections (37%), and negative and mixed infections both accounted for 24% (Figure 2A). CM was primarily composed of negative patients, accounting for a high proportion of 67%, followed by bacterial–fungal co-infections (12%) and bacterial infections (10%) (Figure 2B). However, as presented in Figure 2C, there was a limited concordance between CM and mNGS. Only 22% of the results showed an exact match (Double- and Match), with mNGS+ alone accounting for 46%. Therefore, the distribution of bacterial and fungal detections demonstrated significant differences (Figure 3). In the acute infection group, mNGS detected a more diverse set of bacteria and fungi. CM was dominated by *Acinetobacter baumannii, Klebsiella pneumoniae*, and *Candida albicans*, and mNGS was dominated by *Klebsiella pneumoniae, Prevotella*, and *Candida albicans*. There was a little difference in the distribution of bacteria in the chronic infection group, with both CM and mNGS showing dominance of *Acinetobacter baumannii, Klebsiella pneumoniae*, and *Pseudomonas aeruginosa*. However, mNGS could detect more fungi, with *Candida albicans* and *Candida glabrata* being the predominant species. In contrast, CM was dominated by *Candida albicans*.

**Figure 4:**
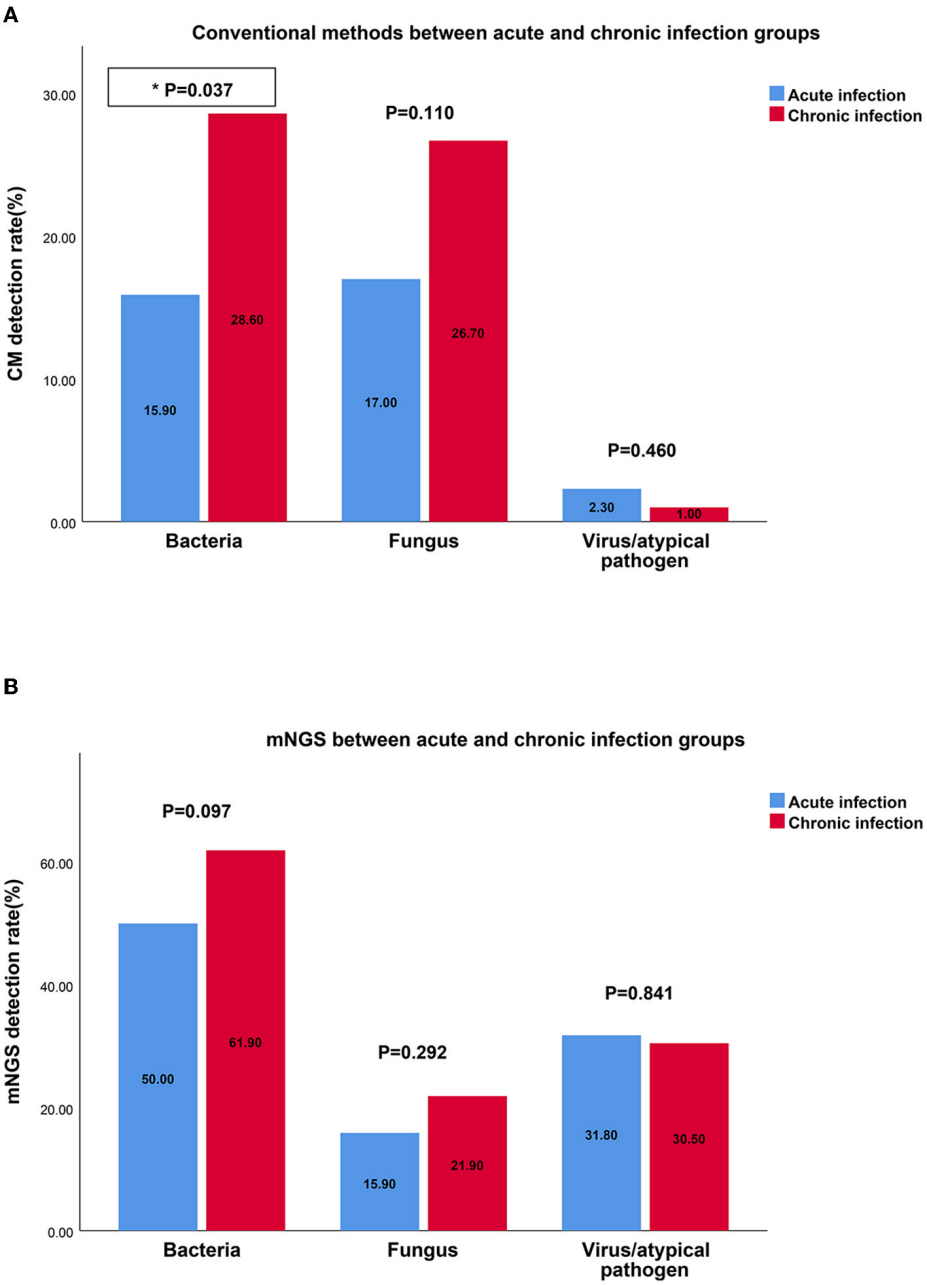
The detection rates of bacteria, fungus, virus by CM and mNGS in different groups. **(A)** The detection rate of bacteria, fungus, virus by CM between acute infection group and chronic infection group; **(B)** The detection rate of bacteria, fungus, virus by mNGS between acute infection group and chronic infection group. mNGS, metagenomic next-generation sequencing; CM, culture methods.

Compared with the chronic infection group, CM exhibited a lower detection rate for bacteria in the acute infection group. However, regardless of the disease type or duration, mNGS demonstrated more consistent results and displayed a stable ability to detect bacteria, fungi, and viruses (Figure 4).

### mNGS is beneficial to medication and rehabilitation

**Figure 5:**
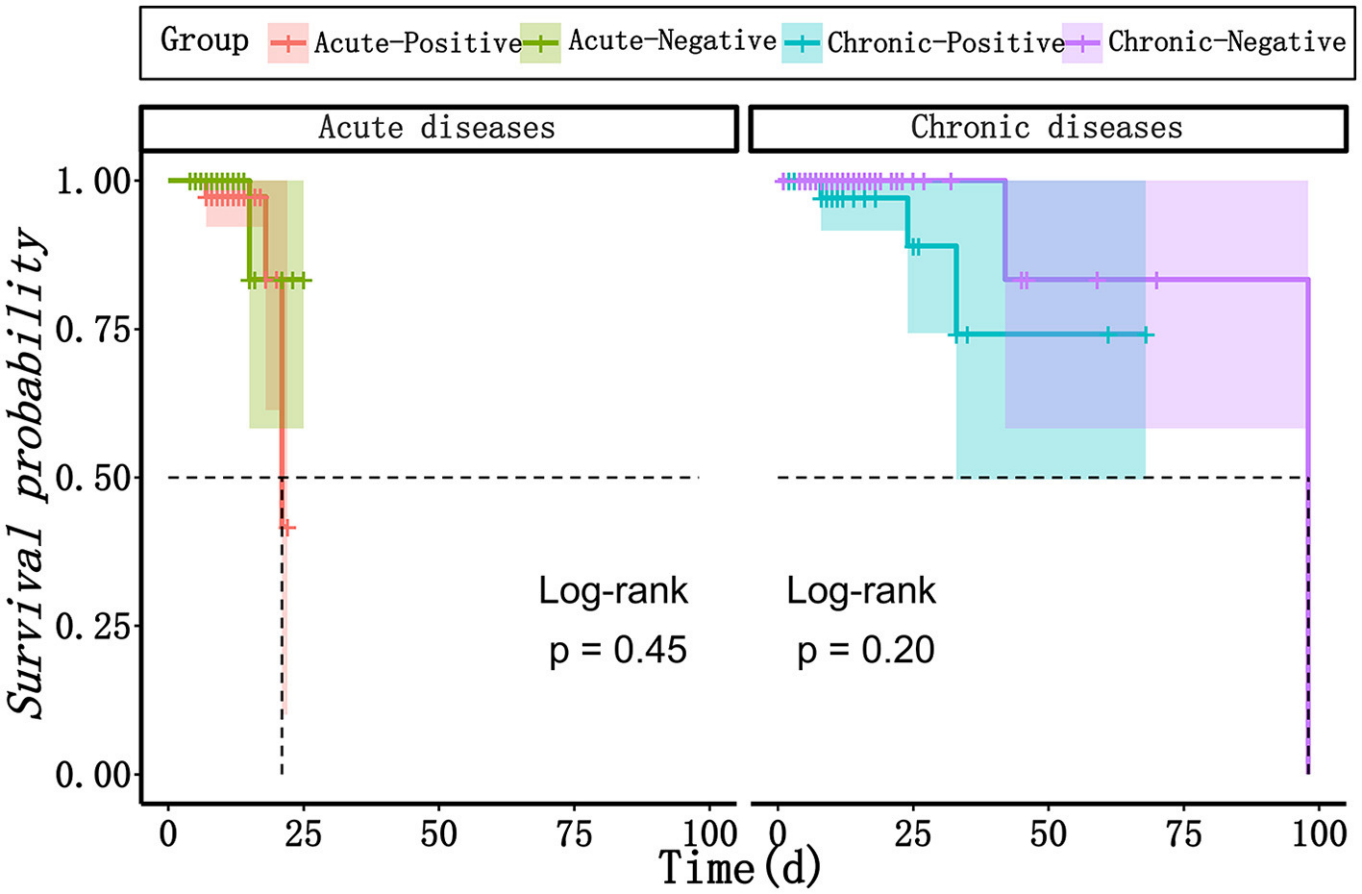
The survival curves between mNGS-positive group and mNGS negative group in patients with acute infection and chronic infection, respectively. mNGS, metagenomic next-generation sequencing.

The results of mNGS are beneficial for clinicians to make decisions for antibiotics, as well as for the decline and recovery of inflammatory indicators in patients with acute infections. As shown in Table 3, in both acute and chronic infection groups, 60.5 and 46.2% of patients adjusted their antibiotic category based on the positive mNGS results, compared with those with negative results (60.5 vs. 28.0%, *P* = 0.002; 46.2 vs. 22.6%, *P* = 0.011). In addition, in the acute infection group, negative mNGS tended to predict the decline in inflammatory indicators, such as the percentage of patients with neutrophils returning to normal could be increased from 64.0 to 94.4% (*P* = 0.028); however, there was no significant benefit of mNGS in the chronic infection group for the recovery of their infection indicators. Moreover, as shown in Figure 5, the results of mNGS could not improve the survival time of the patients, and the COX regression did not show significant effects on survival about age, gender, antibiotics, mNGS, and inflammation indicators (Table 4). Therefore, the results of mNGS detection could be beneficial to clinicians to adjust the use of antibiotics against the detected pathogens and predict the recovery of inflammatory indicators in acute infections.

**Table 3:** Comparisons between acute infection and chronic infection group.

**Variables**	**Acute infection**			**Chronic infection**		
	**Positive (*****n*** = **38)**	**Negative (*****n*** = **50)**	***P***	**Positive (*****n*** = **52)**	**Negative (*****n*** = **53)**	***P***
**Hospital days** (quartile)	9 (6.5, 12)	11 (8, 16)	0.831	10 (6.25, 17.5)	12 (8, 18)	0.311
**Antibiotic**			**0.0022**			**0.0112**
Changed	23 (60.5%)	14 (28.0%)		24 (46.2%)	12 (22.6%)	
Unchanged	15 (39.5%)	36 (72.0%)		28 (53.8%)	41 (77.4%)	
**The decrease of CRP**	*n* = 35	*n* = 25	0.766	*n* = 29	*n* = 24	0.626
Median (min, max)	56.8 (−87.0, 290)	33.3 (−54.0, 619)		25.5 (−44.3, 180)	40.0 (−128, 205)	
**CRP return to normal**	*n* = 35	*n* = 25	0.963	*n* = 29	*n* = 24	0.0959
Yes	11 (31.4%)	8 (32.0%)		5 (17.2%)	9 (37.5%)	
No	24 (68.6%)	17 (68.0%)		24 (82.8%)	15 (62.5%)	
**The decrease of WBC**	*n* = 15	*n* = 13	0.856	*n* = 16	*n* = 14	0.58
Median (min, max)	3.46 (−16.7, 12.0)	3.06 (−18.4, 13.5)		−0.328 (±5.27)	0.897 (±8.80)	
**WBC return to normal**	*n* = 15	*n* = 13	0.705	*n* = 16	*n* = 14	0.491
Yes	8 (53.3%)	6 (46.2%)		6 (37.5%)	7 (50.0%)	
No	7 (46.7%)	7 (53.8%)		10 (62.5%)	7 (50.0%)	
**The decrease of neutrophil**	*n* = 25	*n* = 18	0.331	*n* = 20	*n* = 17	0.351
Median (min, max)	12.4 (−6.30, 82.2)	16.3 (2.00, 54.7)		6.04 (±12.1)	1.92 (±14.1)	
**Neutrophil return to normal**	*n* = 25	*n* = 18	**0.0284**	*n* = 20	*n* = 17	0.969
Yes	16 (64.0%)	17 (94.4%)		6 (30.0%)	5 (29.4%)	
No	9 (36.0%)	1 (5.6%)		14 (70.0%)	12 (70.6%)	

PCT, procalcitonin; CRP, C-reactive protein; WBC, white blood cell; N, neutrophil. The bold values indicate that *p*-value is less than 0.05.

**Table 4:** COX regression analysis between acute infection group and chronic infection group.

**Variables**	**Acute infection group**		**Chronic infection group**	
	**HR (95%CI)**	***P***	**HR (95%CI)**	***P***
Sex		0.372		0.695
Female	Reference		Reference	
Male	2.835 (0.288, 27.890)		0.621 (0.058, 6.700)	
Age		0.994		0.991
< 45	Reference		Reference	
45–60	0 (0, 0)	0.980	6,962.448 (0, 7.210E+125)	
60–75	1.001 (0.090, 11.129)	0.999	1.026 (0, 1.039E+132)	
≥75	0.711 (0.043, 11.639)		97,260.235 (0, 1.001E+126)	
Anti-infective therapy		0.462		0.203
Unchanged	Reference		Reference	
Modified	2.353 (0.240, 23.046)		4.362 (0.451, 42.188)	
NGS		0.997		0.795
No pathogens	Reference		Reference	
Bacteria	0.237 (0.009, 6.332)	0.391	3,650.339 (0, 3.148E+47)	0.875
Fungus	0 (0, 0)	0.998	0.007 (0, 0)	0.998
Virus/atypical pathogen	0 (0, 0)	0.984	2,245.673 (0, 1.937E+47)	0.881
Bacteria + fungus	0.618 (0.025, 15.449)	0.769	0.008 (0, 0)	0.996
Bacteria + virus/atypical pathogen	0 (0, 0)	0.993	1.072 (0, 1.045E+62)	0.999
Fungus + virus/atypical pathogen	0.371 (0.011, 12.584)	0.582	33,627.634 (0, 2.906E+48)	0.840
Bacteria + fungus + virus/atypical	0 (0, 0)	0.997	0.008 (0, 0)	0.998
**Pathogen**				
mNGS		0.457		0.229
Negative	Reference		Reference	
Positive	2.409 (0.237, 24.488)		4.177 (0.407, 42.841)	
PCT		0.800		0.795
< 0.5	Reference		Reference	
≥0.5	0.774 (0.107, 5.604)		0.039 (0, 1518968057)	
CRP		0.784		0.663
< 10	Reference		Reference	
≥10	26.132 (0.000, 3.684E+11)		29.576 (0, 32876025.15)	
WBC		0.637		0.959
< 10	Reference		Reference	
≥10	1.484 (0.287, 7.661)		0.938 (0.081, 10.848)	
N%		0.933		0.396
< 75	Reference		Reference	
≥75	1.101 (0.117, 10.379)		58.127 (0.005, 692682.844)	
L%		0.638		0.535
< 20	Reference		Reference	
≥20	1.517 (0.267, 8.606)		0.029 (0, 2040.882)	

mNGS, metagenomic next-generation sequencing; CM, culture methods; WBC, white blood cell; PCT, procalcitonin; CRP, C-reactiveprotein; N, neutrophil; L, lymphocyte; HR, hazard ratio; CI, confidence interval.

### Recommended mNGS for patients with acute infections

**Figure 6:**
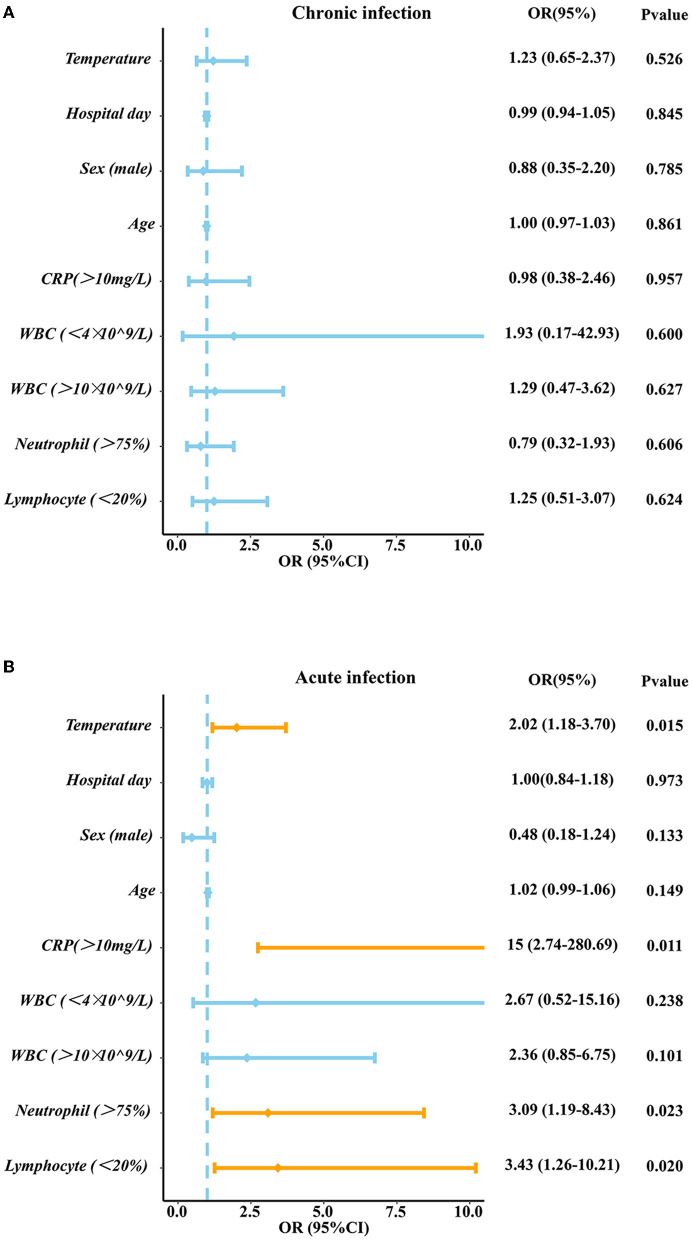
The Logistic analysis in acute infection group and chronic infection group. **(A)** The Logistic analysis in chronic infection group **(B)** The Logistic analysis in acute infection group. PCT, procalcitonin; CRP, C-reactive protein; WBC, white blood cell; N, neutrophil; L, lymphocyte.

**Figure 7:**
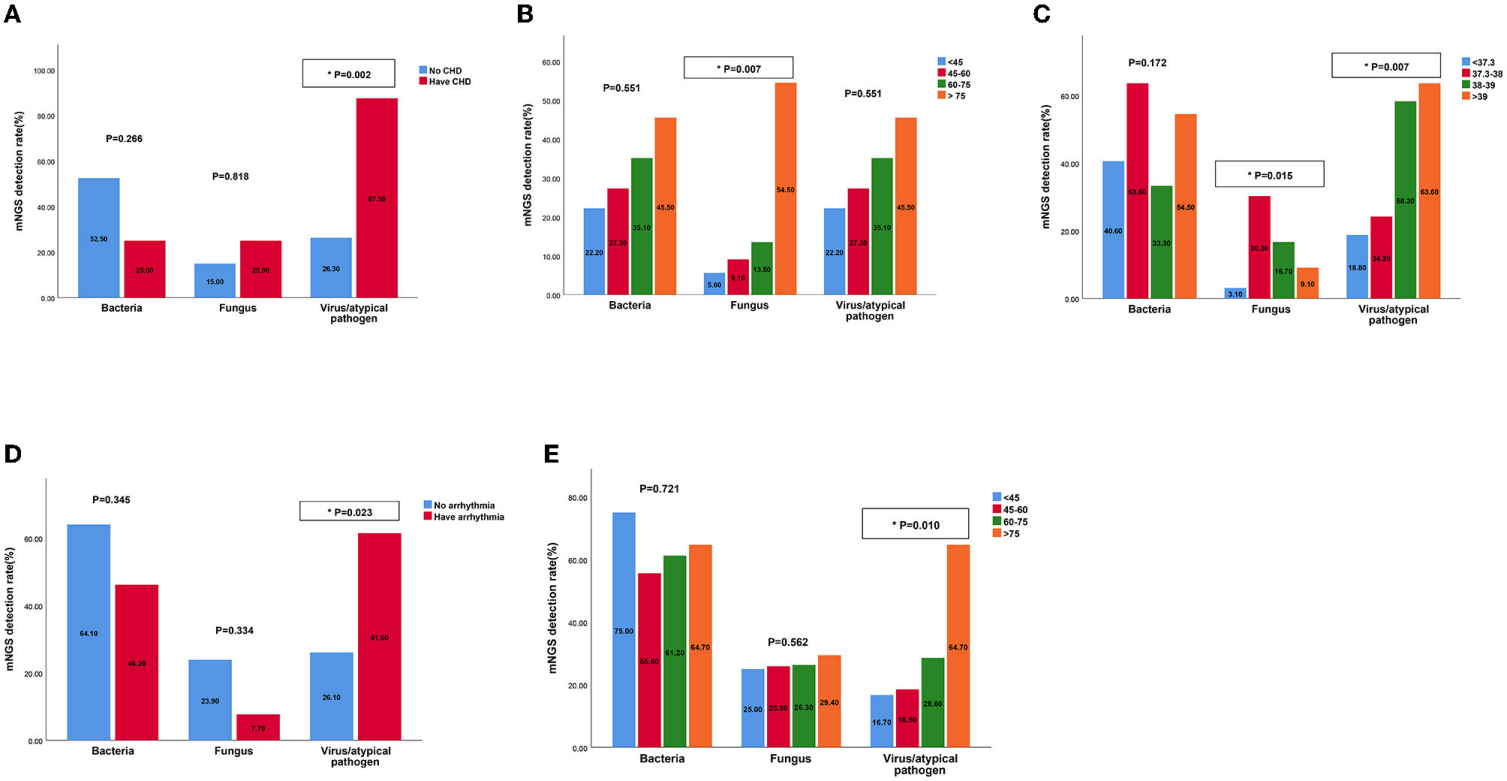
The detection rates of bacteria, fungus, virus by mNGS in patients with different characteristics of acute and chronic infection groups. **(A)** The detection rates of bacteria, fungus, virus by mNGS in patients with CHD in acute infection group; **(B)** The detection rates of bacteria, fungus, virus by mNGS in patients of different ages in acute infection group; **(C)** The detection rates of bacteria, fungus, virus by mNGS in patients with different temperatures in acute infection group; **(D)** The detection rates of bacteria, fungus, virus by mNGS in patients with arrhythmia in chronic infection group; **(E)** The detection rates of bacteria, fungus, virus by mNGS in patients of different ages in chronic infection group. mNGS, metagenomic next-generation sequencing; CHD, cornary heart diseases.

We predicted the potential factors which may lead to the positive mNGS results in the acute or chronic infection groups through regression analysis. As shown in Figure 6, pathogenic microorganisms in the acute infection group were more likely to be detected by mNGS if the patients were found to have fever, C-reactive protein (CRP) > 10 mg/L, neutrophil > 75%, or lymphocyte < 20%. On the contrary, abnormalities in the above indicators in the chronic infection group could not be used to predict the positive mNGS results. In addition, as shown in Figure 7, viruses or other pathogens were more likely to be detected by mNGS in patients with coronary artery disease or arrhythmia or a high fever (T > 38°C) in the both acute and chronic infection groups (Figures 7A, C, D), whereas fungi were more likely to be detected in patients with a slight fever (37.3–38°C) in the acute infection group (Figure 7C). Furthermore, the acute infection group tended to have an increasing detection rate with fungus, especially for elderly (Figure 7B). In contrast, only viral infections were associated with elderly in the chronic infection group (Figure 7E). Therefore, mNGS was more recommended for elderly with acute infections and abnormal inflammatory markers.

## Discussion

In this study, we compared the test results and clinical features of mNGS and CM for BAL samples between acute infection and chronic infection patients systematically, and the results suggest that mNGS had more advantages in some aspects as compared with CM. Firstly, mNGS is more stable, sensitive and accurate than CM in the detection of bacteria, fungi, viruses and atypical pathogens. mNGS could identify different dominant pathogens in patients with different underlying diseases and clinical characteristics in both acute and chronic infection patients. Secondly, positive mNGS results were more helpful in changed antibiotic therapy for patients with acute infections than those for patients with chronic infections, with results suggesting more pronounced improvement of patients with acute infections. Finally, we found the related risk inflammatory factors of positive mNGS results in acute infection patients.

As a transformational and advanced technology, mNGS is able to detect a wide range of direct and potential infectious pathogens by sequencing of the extracted DNA from different specimens, and has the advantages of unbiased pathogen detection and short detection time (Finotello et al., 2018). Our study showed that the sensitivity, specificity, PPV and NPV were all higher than those of CM, especially the sensitivity, which is similar to some previously published research (Li et al., 2018; Cai et al., 2020). The high sensitivity of mNGS may be attributed to the long survival time of pathogenic genes in BALF, high detection rates of pathogens, and the small impact of antibiotic use on mNGS as compared with that in CM (Gosiewski et al., 2017). In addition, higher sensitivity contributed to higher detection rates of pathogens. Consistent with previous studies (Takeuchi et al., 2019; Ding et al., 2020; Huang et al., 2021), mNGS showed advantages in diagnosis of mixed infection of bacteria, fungi and viruses when compared to CM. Therefore, the relatively low matching rate between mNGS and CM may be attributed to the narrow detection range and low positive rate of CM. However, mNGS had more false-positive results than CM because mNGS could detect oral flora and colonizers in BALF from respiratory tract more easily. Sometimes, results of mNGS may blunt the diagnosis of pathogenicity resulting in the inability to distinguish among microbial infections, colonization and contaminations (Simner et al., 2018). Therefore, clinicians should interpret the mNGS results carefully when confronted with the inconsistent results of conventional methods after considering clinical manifestations and examination results of patients.

When it comes to the guidance of mNGS for clinical practice, we found that there were significantly more patients changed the antibiotic treatments in the positive group than negative group both in acute infection patients (60.5 vs. 28.0%, *P* = 0.0022) and chronic infection patients (46.2 vs. 22.6%, *P* = 0.01112). Consistently, a retrospective study enrolled 130 patients with acute respiratory failure mostly caused by pulmonary infection, and found that 58.5% of these patients had changed antibiotic regimen according to mNGS (Huang et al., 2021). For mNGS-positive patients where the CM was inconclusive, another study found that 58% of their patients were not covered by empirical antibiotics, and 61% of their patients modified antibiotic therapy based on mNGS (Miao et al., 2018). Some recent studies have demonstrated that mNGS has promising advantages in detecting antibiotic resistance genes (Ruppé et al., 2017; Chen et al., 2021a). Therefore, the result of mNGS have been an essential reference to aid clinicians with disease diagnosis and targeted therapeutic schedule. However, there is no benefit for the survival prognosis after the change of antibiotics. In addition, survival analysis of our study showed no differences in the survival rate between positive group and negative groups whether in acute or chronic infection patients, and mNGS positive results had no significant impact on survival outcomes of these patients. A retrospective study enrolling 109 patients with infectious disease or not and collecting different samples including BALF reported that patients with positive mNGS results had higher 28-day mortality than those with negative mNGS results (9.0 vs. 0%, *P* = 0.049), but there was no significant difference in overall survival time, which is partly consistent with our view (Duan et al., 2021). But another retrospective study drew different conclusions that the average time of intensive care unit stay [β, −8.689 (95% CI, −16.176, −1.202); *P* = 0.026] and the time from onset to sequencing [β, −5.816 (95% CI, −9.936, −1.696); *P* = 0.007] of the mNGS-positive group were significantly shorter than those of the mNGS- negative group after analyzing 63 blood samples of critically ill patients, and more patients in mNGS-positive group changed the antibiotic treatment regimen after mNGS [OR, 3.789 (95% CI, 1.176, 12.211); *P* < 0.001] (Geng et al., 2021), which is consistent with the conclusion of another study enrolling 56 ICU patients with 131 samples including BALF (Liang et al., 2023). The reason for this inconsistency may be attributed to the differences of the severity of diseases. It was identified that mNGS was associated with a better diagnosis, treatments and prognosis of infectious patients, especially those critically ill patients with acute respiratory failure (Zhang et al., 2020; Xi et al., 2022). Therefore, mNGS could serve as a novel technology for infectious disease diagnosis and provide useful guidance on antibiotic strategies based on appropriate patient selection and scientific data analysis. Large-scale and prospective studies are required in future to verify the value and impact of mNGS-guided treatments in clinical practice.

Based on the advantages and clinical guidance of mNGS, we then investigated the related factors contributing to the positive mNGS results. It was found that high levels of temperature, CRP, neutrophil and low levels of lymphocyte may lead to the positive mNGS results in acute infection patients while no variable was identified to predict positive results in chronic infection patients. These laboratory indicators will help clinicians make decisions about the utilization of the mNGS. What's more, other characteristics of patients such as comorbidities and age were also identified to predict results of mNGS. Our study demonstrated that viral/atypical pathogens were more likely to be detected in acute and chronic infection patients complicated with heart diseases, which maybe because virus can replicate in cardiomyocyte, leading to heart dysfunction (Kenney et al., 2022). Differently, another study reported that APACHE II score (OR = 1.096), immune-related diseases (OR = 6.544), and hypertension (OR = 2.819) were considered as positive independent factors for mNGS positove results in patients with sepsis infection (Sun et al., 2022). We also found fungi were more likely to be detected in old aged patients in acute infection group, while viral/atypical pathogens were more likely to be detected in such patients of chronic infection group, probasbly because most elderly patients have immunity suppression, antibiotic resistance and comorbidity with other diseases (Aronen et al., 2019; Chen et al., 2021b). Similar to our study, a study used mNGS from plasma samples to dignose 40 travelers with acute fever (≥38°C), and found 11 patients were diagnosed with viral infection, highlighting the diagnostic value for acute high fever patients (Jerome et al., 2019).

Therefore, mNGS proved to be more valuable to acute infection patients than chronic infection patients. Various studies have proved the clinical value of mNGS for acute infectious patients. Some studies discussed the application of mNGS in acute viral encephalitis (Cao and Zhu, 2020), acute respiratory distress syndrome (Zhang et al., 2021; Wang et al., 2022), acute respiratory distress syndrome (Zhang et al., 2023) and other acute infectious diseases, demonstrating that mNGS is a promising tool for the diagnosis of acute disease caused by multiple infectious agents. mNGS represents a valuable supplementary tool to CM in order to rapidly determine etiological factors of various infections and guide treatment decision- making for patients (Xu et al., 2023). However, mNGS will interfere with the diagnosis of pathogenic bacteria when detecting broad-spectrum pathogens, resulting in an inability to distinguish among microbial infections, colonization and contaminations. The cost and quality surveillance of mNGS still need more efforts. Therefore, further studies are required to not only identify the complementary role of mNGS from different samples in order to support CM in routine clinical practice (Gu et al., 2021), but also explore more rapid, economic and targeted technology of mNGS to achieve early and accurate diagnosis of infectious diseases (Li et al., 2022).

In this study, we compared mNGS and CM in sensitivity, specificity, and pathogen types. On this basis, we also compared and analyzed the differences between the positive and negative groups of mNGS between acute and chronic infection patients in multicenters for the first time. Higher diagnostic value of metagenomic next- generation sequencing was demonstrated in patients with acute infection than patients with chronic infection in our study. However, the sample size in this study is relatively small. There is also a lack of randomized controls. And the retrospective nature of the study may miss some important data, which may bias the results and conclusion. Finally, the lack of a gold standard comparator for diagnostics, classification bias and antibiotic usage details may limit the generalizability of the conclusion of the present study.

## Data availability statement

The original contributions presented in the study are included in the article/Supplementary material, further inquiries can be directed to the corresponding authors.

## Ethics statement

The studies involving humans were approved by the Ethics Committee of the 10th People's Hospital affiliated to Tongji University and Tongren Hospital. The studies were conducted in accordance with the local legislation and institutional requirements. The human samples used in this study were acquired from a by-product of routine care or industry. Written informed consent for participation was not required from the participants or the participants' legal guardians/next of kin in accordance with the national legislation and institutional requirements.

## Author contributions

AY: Formal analysis, Investigation, Methodology, Software, Supervision, Writing—original draft, Writing—review & editing. JW: Formal analysis, Methodology, Software, Writing—original draft. QX: Data curation, Formal analysis, Writing—original draft. BS: Data curation, Formal analysis, Writing—original draft. KS: Data curation, Writing—review & editing. FH: Data curation, Supervision, Validation, and Writing—review & editing. CW: Conceptualization, Data curation, Funding acquisition, Resources, Supervision, Validation, Writing—review & editing. SX: Conceptualization, Data curation, Funding acquisition, Resources, Supervision, Validation, Writing—review & editing.
