# Supplementary figures and images for: Higher diagnostic value of metagenomic next-generation sequencing in acute infection than chronic infection: a multicenter retrospective study
Source: Front Microbiol. 2024 Jan 29;15:1295184. doi: 10.3389/fmicb.2024.1295184 (PMC10864100; doi:10.3389/fmicb.2024.1295184)

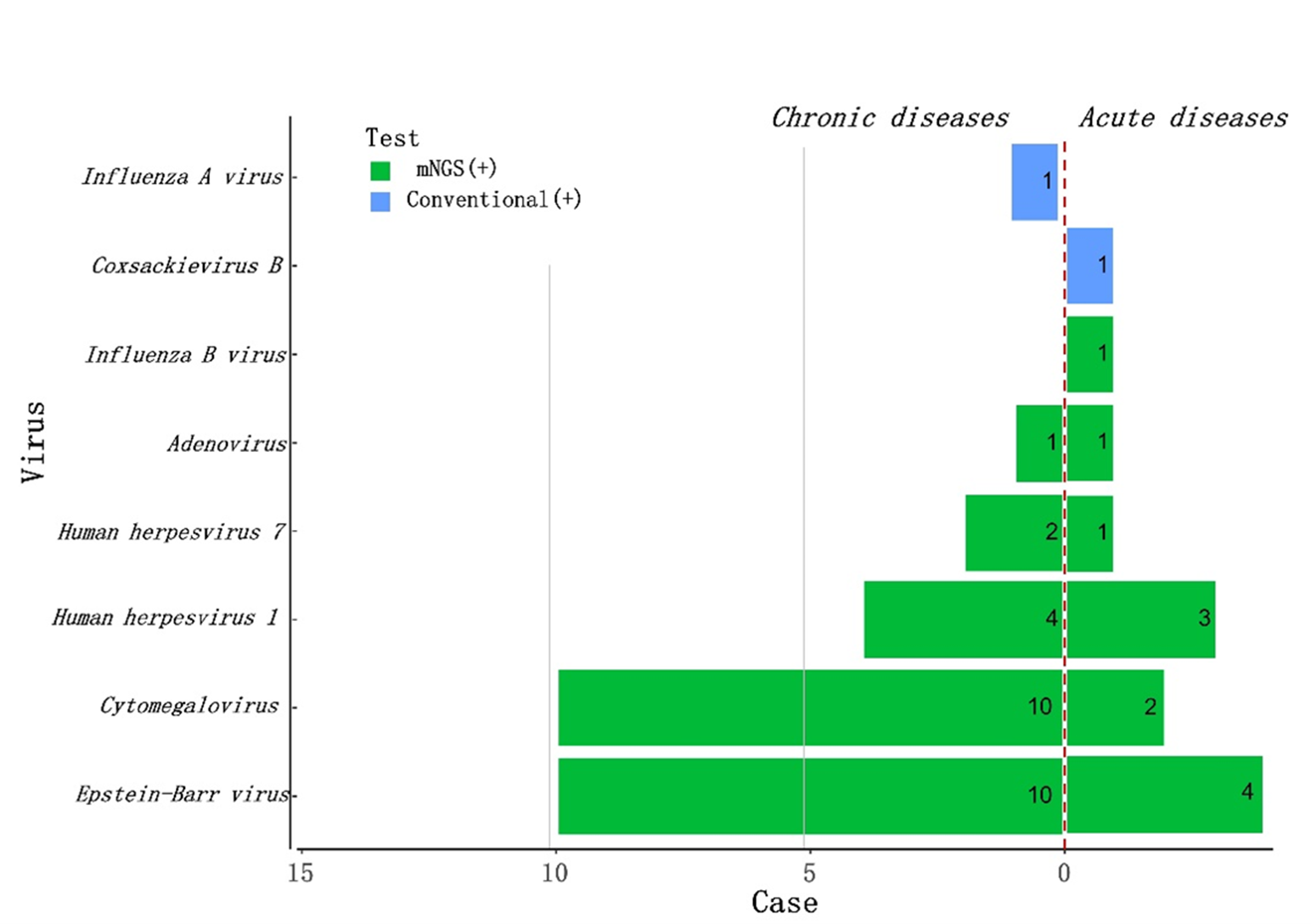

Supplement: Supplementary file 3 [file Image_1.TIF]

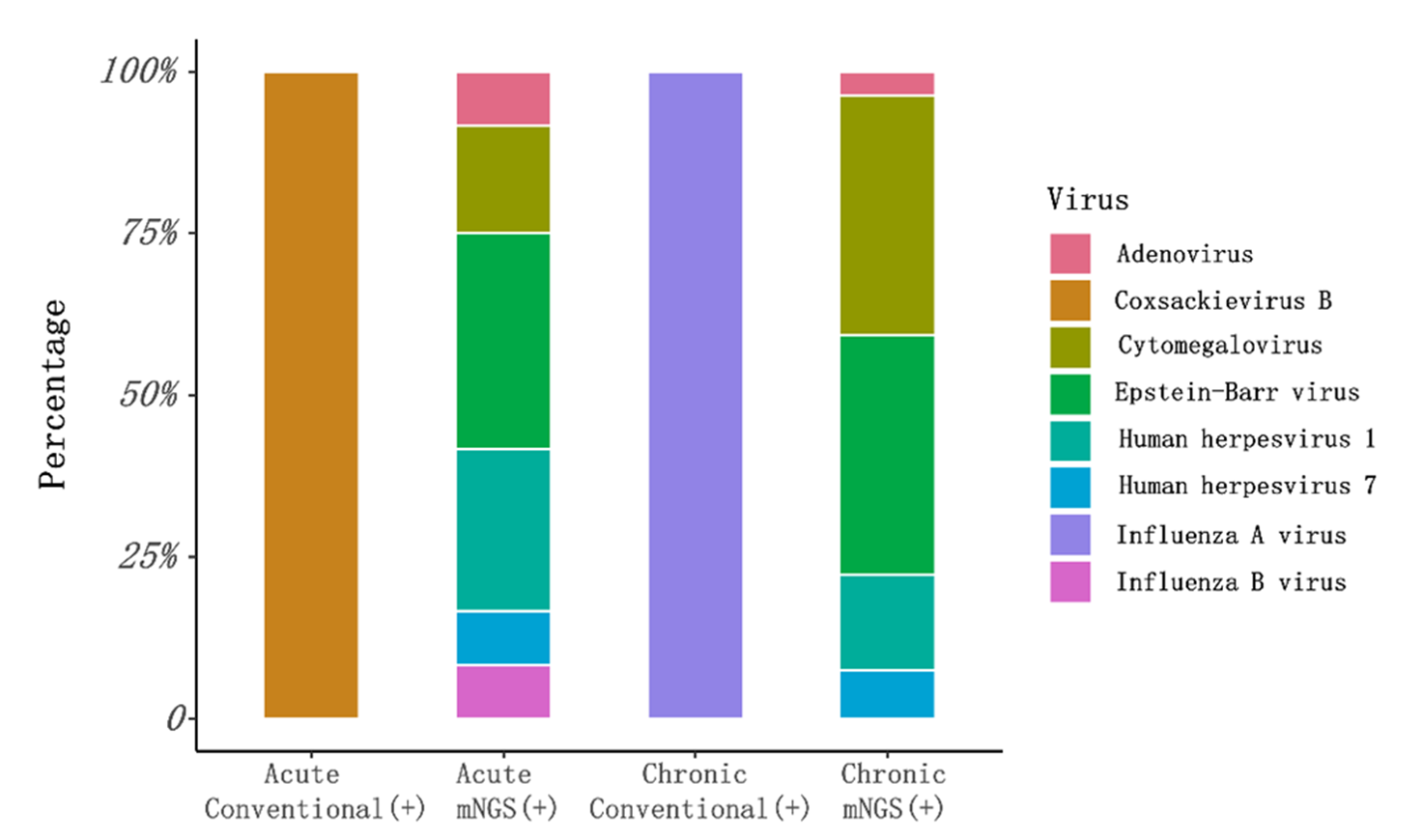

Supplement: Supplementary file 4 [file Image_2.TIF]
